# Management of foetal asphyxia by intrauterine foetal resuscitation

**DOI:** 10.4103/0019-5049.71032

**Published:** 2010

**Authors:** S. Velayudhareddy, H Kirankumar

**Affiliations:** Department of Anaesthesiology, Rajeev Gandhi Institute of Medical Sciences, Kadapa, Andhra Pradesh, India

**Keywords:** Foetal asphyxia, anaesthesiologist, resuscitation, management

## Abstract

Management of foetal distress is a subject of gynaecological interest, but an anaesthesiologist should know about resuscitation, because he should be able to treat the patient, whenever he is directly involved in managing the parturient patient during labour analgesia and before an emergency operative delivery. Progressive asphyxia is known as foetal distress; the foetus does not breathe directly from the atmosphere, but depends on maternal circulation for its oxygen requirement. The oxygen delivery to the foetus depends on the placental (maternal side), placental transfer and foetal circulation. Oxygen transport to the foetus is reduced physiologically during uterine contractions in labour. Significant impairment of oxygen transport to the foetus, either temporary or permanent may cause foetal distress, resulting in progressive hypoxia and acidosis. Intrauterine foetal resuscitation comprises of applying measures to a mother in active labour, with the intention of improving oxygen delivery to the distressed foetus to the base line, if the placenta is functioning normally. These measures include left lateral recumbent position, high flow oxygen administration, tocolysis to reduce uterine contractions, rapid intravenous fluid administration, vasopressors for correction of maternal hypotension and amnioinfusion for improving uterine blood flow. Intrauterine Foetal Resuscitation measures are easy to perform and do not require extensive resources, but the results are encouraging in improving the foetal well-being. The anaesthesiologist plays a major role in the application of intrauterine foetal resuscitation measures.

## INTRODUCTION

Intrauterine foetal resuscitation (IUFR) initially developed in 1960. The goal of intrauterine foetal resuscitation was to optimize the foetal condition *in utero*, so that labour may continue safely for normal delivery or improve foetal the well-being prior to emergency operative delivery. IUFR describes the application of measures to a mother in active labour with foetal distress, with the intention of improving oxygen delivery to the compromised foetus. IUFR measures are easy to perform, and can result in significant improvement in the foetal condition. Despite these advantages they are not widely used by obstetricians. A compliance report from the American College of Obstetricians and Gynaecologists show that only 16% of the patients received tocolysis for foetal distress.[1] An audit of IUFR in the UK centre revealed that complete IUFR measures were applied in only 20% of patients having caesarian section for foetal distress.[2] However, in their absence anaesthesiologists still use IUER measures, when they are directly involved in managing a woman in active labour, for example, Labour analgesia and before operative delivery.

## FOETAL DISTRESS DEFINITION

Foetal distress is an imprecise and non-specific term used to describe a non-reassuring foetal heart trace with or without conformation of foetal acidosis from a foetal blood sample (FBS). Parer and Livingston[3] described foetal distress as, ‘progressive foetal asphyxia that if not corrected or circumvented, will result in decompensation of the physiological response (primarily redistribution of blood to preserve oxygenation to vital organs) and cause permanent central nervous system damage and other damage or death.’ In UK, the foetal heart rate pattern is classified as normal, abnormal and suspicious[4] on the basis of the appearance of the foetal heart. A suspicious (or) abnormal foetal heart may indicate the presence of foetal hypoxia and acidosis, but this is not universal. In this situation the foetal blood sample is advised, to accurately assess the foetal metabolic state and to guide for further management.[4] However, collecting a foetal blood sample may be difficult and time consuming and in some cases fails to yield any result,[5] necessitating an operative delivery on the basis of foetal heart rate only.

## CONDITIONS CAUSING FOETAL DISTRESS

Acute deterioration of a foetal heart pattern may be caused by two types of factors (I) Transient factors like increased uterine activity, poor maternal position, maternal hypotension, or cord compression during uterine contractions. (II) Permanent factors or Irreversible factors such as placental abruption, maternal or foetal haemorrhage, uterine rupture, or cord prolapse. Unfortunately foetal heart monitoring cannot reliably distinguish between these two situations. The clinical situation has to be reviewed continuously and carefully after deterioration in the foetal heart rate pattern, and intrauterine foetal resuscitation measures should be initiated whenever necessary; there is a potential for a spectrum of outcome from complete recovery to foetal death. The use of IUFR is based on the understanding of physiology and oxygen transfer and effect of uterine contractions.

## DIAGNOSIS

Foetal heart rate (FHR) monitoring during labour can give us an indication of foetal hypoxia and acidity. When FHR tracing measures as normal there is a predictive value of 99% for confirming a non-acidic foetus, and an abnormal FHR tracing has a positive predictive value of 50% for foetal compromise.[6] Measurement of intrapartum acid base status obtained from foetal scalp blood helps to decrease operative deliveries following false positive foetal heart tracings.[7] Several methods of continuous blood gas monitoring using an electrode attached to the foetal scalp sub or transcutaneous has been tried for PO_2_, PCO_2_, PH. Although foetal scalp sampling[8] has a greater specificity than FHR monitoring alone, it is rarely used in many institutions because of its invasive nature, unreliable results and procedure requirement.[9] Foetal scalp or acoustic stimulation[10] has also been used, but the results are not satisfactory. Foetal pulse oxymetry (FPO)[11] was proposed as an adjuvant to conventional electronic foetal monitoring (EFM). Bloom and others[12] in their study found potential side effects of such monitoring in both mother and neonate. Thus they discontinued this diagnostic tool.

## PHYSIOLOGY OF FOETAL CIRCULATION AND OXYGEN DELIVERY

At term, umbilical venous PO_2_ is 30 – 35 mm Hg despite the low umbilical venous PO_2,_ the foetal haemoglobin is 75 – 80% saturated because of the number of factors. A high foetal haematocrit (17 – 19 g/dl), high cardiac index (350 mls/m^2^/min) and the presence of foetal haemoglobin F helps to ensure adequate oxygen content. Oxygen carriage to vital organs is also dependent on foetal cardiac output and adequate umbilical circulation. Stroke volume is relatively fixed and FHR is the major determinant of cardiac output. The foetal circulation is also designed such that the best oxygenated blood from the umbilical vein is directed via the ductus venosus to the inferior vena cava and then to the left side of heart and then to the head and neck of the foetus. The less oxygenated blood from the superior vena cava enters the right ventricle and aorta via the ductus arteriosus, to distribute to the trunk and lower part of body, of the foetus.

## UTERINE OXYGEN DELIVERY

Uterine oxygen delivery is defined as the product of organ blood flow and oxygen content of the arterial blood. Placental blood flow is determined by uterine perfusion pressure (Arterial pressure – Venous pressure) and the resistance to blood flow. Oxygen delivery is defined as the placental blood flow multiplied by the arterial oxygen content (Haemoglobin concentration multiplied by oxygen saturation). Branches of uterine arteries supply the blood to the intervillous space and the blood returns to the maternal circulation via the uterine veins. The oxygen delivery is close to maximum at term provided that the mother has normal haemoglobin, normal oxygen saturation and perfusion pressure.

## EFFECT OF UTERINE CONTRACTIONS ON OXYGEN TRANSPORT

In an uncomplicated pregnancy the uterine spiral arteries are maximally vasodilated during late pregnancy and oxygen delivery to the placenta is close to maximum. During labour, the active uterine contractions increase the intrauterine pressure up to 45 – 50 mm Hg, which initially compresses the veins, so intervillous blood volume increases until the intrauterine pressure is sufficient to stop the uterine arterial blood flow. This causes a reduction in the PO_2_ of the blood in the intervillous space and the foetal oxygen saturation declines to about 7%, to a low point, at about 90 – 120 seconds after the peak contraction. Recovery occurs over a similar period of time. When oxygen delivery is border line, the contraction may cause a marked fall in oxygen saturation and foetal bradycardia. When the oxygen delivery is severely impaired oxygenation fails to recover between contractions and sustained bradycardia results.

## AORTOCAVAL COMPRESSION

The pregnant uterus usually at term can compress the inferior vena cava and descending aorta within the abdomen, resulting in hypotension, reduced uteroplacental perfusion pressure and blood flow. The effect is maximal in the supine poison, but can also occur to a lesser extent in the supine position with tilt, semi-recumbent position and even in the standing position.[13] Maternal changes associated with systemic hypotension will usually ensure a corrective change in position, but Aortocaval compression can be asymptomatic and foetal compromise may go undetected. Neonatal outcome is better if the second stage of labour is managed with the addition of pelvic tilt to supine position.

## PLACENTAL OXYGEN TRANSPORT

The placental chorionic villi project into the large lakes of maternal blood in the intervillus space and contain foetal capillaries. These chorionic villi are perfused by the umbilical arteries and the blood returns to the foetal circulation via the umbilical vein. The placental transfer of oxygen is a passive process and oxygen transport occurs along a gradient from a high maternal PO_2_ of 100 mmHg to a lower foetal PO_2_ of 35 mmHg. Foetal haemoglobin has a much greater affinity for oxygen and lower affinity for 2, 3-diphosphoglycerate than adult haemoglobin and this facilitates oxygen transfer. Dissolved carbon dioxide passes from the foetal to maternal blood causing reduced affinity of maternal haemoglobin for oxygen and increases the affinity of foetal haemoglobin for oxygen. This is called the ‘Double Bore Effect’ and accounts for 80% of the oxygen transfer. During uterine contractions the umbilical blood flow usually continues during the period of static intervillous flow, resulting in gradual reduction in the foetal oxygen uptake from the intervillous space, causing a decline in the foetal oxygen saturation by 7%, during uterine contractions. If the mother breathes 100% oxygen at that time, maternal oxygen partial pressure increases to the maximum, and foetal oxygen saturation increases by 34% and partial pressure by 68%.[13] An increase in foetal oxygen saturation starts within minutes of administration of oxygen to the mother, but the peak level may not be reached until 10 minutes.

## IUFR MEASURES

The IUFR consists of specific measures aimed to increase the delivery of oxygen to the placental and umbilical blood flow, in order to reverse foetal hypoxia and acidosis. The mother should be examined quickly to exclude maternal hypoxia or shock or placental separation, which requires specific additional therapy and the IUFR measures should be instituted immediately [Table 1].

**Table 1:** Intrauterine foetal resuscitation measures

Maternal position	Left lateral position, Right lateral, or knee elbow position (in case of cord compression)
Tocolysis	Turn off syntocinon drip
	Terbutaline 250 micrograms S/C or I.V, or GNT spray sublingually — two puffs, can be repeated thrice
Oxygen administration	10 to 15 litres / minute by tightly fitting and non-rebreathing Hudson’s face mask
Rapid intravenous fluids	One litre of crystalloid, Hartmen’s solution or normal saline rapidly
Vasopressors	Ephedrine, consider during maternal hypotension
Amino-infusion	Infuse 250 to 500 ml of normal saline, monitoring FHR and measuring uterine baseline tone. (oligohydramnios, vaginal leak)

## MATERNAL POSITION

Changing the maternal position from supine to left lateral position relieves the Aortocaval compression and improves the FHR abnormalities. These outcomes correlate with improved foetal oxygen saturation. This improvement occurs even in the absence of maternal hypotension, as aortic compression occurs independently of vena cava compression. The left lateral position is used in IUFR, because the highest cardiac output is achieved in this position. However, if there is no improvement in FHR with left lateral position, the right lateral or knee elbow position should be tried. The Royal College of Obstetricians and Gynaecologists Guidelines (RCOG) on electronic foetal monitoring, recommended the use of the left lateral position in any case of suspected foetal distress, and the FBS sampling should be taken in the left lateral position.[4]

## TOCOLYSIS

Tocolysis is the procedure used for the active management of foetal distress, when FHR abnormality occurs in the presence of any uterine activity. Tocolysis improves uteroplacental perfusion and reduces cord compression (if present) by decreasing the baseline tone of the uterus, which is the main cause for FHR abnormalities. The RCOG guidelines suggest Tocolysis with Terbutaline 250 mg subcutaneously, to reduce uterine contraction frequency and baseline tone.[4] Terbutaline may increase the maternal heart rate, but does not increase the cardiovascular side effects during anaesthesia. Thurlow JA, Kinsella SM used sublingual Glyceryl trinitrate 0.8 mg for tocolysis, which showed a decrease in baseline uterine tone and improvement in foetal heart rate.[14] Atosiban is an oxytocine receptor antagonist that has a high specificity for stopping pre-term labour. Its use for foetal distress has been evaluated in a pilot study. In comparison with hexoprenaline, Atosiban was as effective at stopping uterine contractions, but with a shorter duration and fewer maternal side effects.[15]

## MATERNAL OXYGEN ADMINISTRATION

Prolonged administration of oxygen is not advisable as per ROCG guidelines, but there is insufficient evidence to evaluate the short-term use of oxygen in case of foetal distress.[4] Anyway administration of FiO2 of 1.0% of oxygen to the mother increases the foetal oxygen concentration, but the time required to achieve the maximum foetal oxygen concentration will be increased in the presence of foetal distress. This effect is not reliable with a FiO_2_ of 0.4%, but it is difficult to deliver FiO2 of 1.0% without an anaesthesia circuit. However, Boumphery *et al*.[16] have shown that using a Hudson non-rebreathing mask with three valves, oxygen flow of 15 litres/minute and fitting the mask tightly to the face should achieve an average FiO_2_ of 0.97%.

## RAPID INTRAVENOUS FLUID

Rapid intravenous infusion of one litre of crystalloid solution is recommended in case of foetal distress,[17] as there is no auto-regulation to the uterus. The uterine blood flow will be directly related to the maternal blood pressure. In the presence of maternal hypotension, the fluid infusion will improve the venous return, and thus, the cardiac output. If hypovolaemia due to haemorrhage is the cause of maternal hypotension, blood products should be considered in addition to fluid resuscitation. Even though there is no maternal hypotension, fluid infusion shows improvement in the foetal condition. This improvement is most probably because of reduction in uterine contractions and blood viscosity. The use of intravenous fluids for IUFR is not recommended in the case of ‘pre- eclampsia’.

## VASOPRESSORS FOR CORRECTION OF MATERNAL HYPOTENSION

If an acute deterioration of FHR occurs due to maternal hypotension during labour, or during regional analgesia, instead of all the above-mentioned measures vasopressors should be considered, to restore the cardiac output and maternal blood pressure. Ephedrine crosses the placental and has direct metabolic effects on the foetus as shown by the lower umbilical artery PH, in comparison to phenylephrin. Ephedrine increases the FHR[18] when used during labour. The foetal cardiac output depends on FHR, and therefore, in the situation of foetal bradycardia, the benefit of an increase in FHR is likely to outweigh the deleterious metabolic effects of Ephedrine.

## AMNIOINFUSION

Amnioinfusion prevents or relieves umbilical cord compression usually caused by oligohydramnios. Amnioinfusion has been shown to reduce the incidence of variable foetal heart deceleration. In a randomized prospective study by Mino *et al*.[19] 200 term pregnancies with low amniotic fluid due to vaginal loss were randomly studied, with amniotic infusion and without amniotic infusion, and it was found that amnioinfusion improved the foetal heart rate pattern, lowered operative delivery and improved neonatal acid base status.

In the procedure of amnioinfusion, informed consent is obtained and vaginal examination is performed to evaluate for cord prolapse, establish dilatation and confirm presentation. The foetal scalp electrode is placed, followed by an intrauterine pressure catheter to document the resting tone (< 15 mm Hg). Normal saline is linked to the intravenous tubing, and it is started as it would be for intravenous use, and it is then inserted into the infusion port on the three-way stop cock of the intrauterine pressure catheter. Recommendations for infusion protocols may vary by institution. A common protocol starts with an initial bolus of 250 ml infused for over 20 to 30 minutes. The rate is then adjusted according to the severity of decelerations, but usually at a rate of 10 to 20 ml per minute up to 600 ml, or to resolution of the variable decelerations. An additional 250 ml beyond the volume at which decelerations resolve is administered, then the infusion is terminated, unless the decelerations resume. It is considered a failure if 800 to 1,000 ml of saline does not result in the termination of decelerations. The foetal heart rate and resting tone are assessed continuously during the intervention. If the uterine tone is persistently elevated, the infusion is discontinued, and the uterine pressure is allowed equilibrating. The resting uterine tone is reassessed, and if the new resting tone is 15 mm Hg above the baseline resting tone or a maximum of 30 mm Hg the infusion is discontinued.

## ANAESTHESIA

The national Sentinel Audit of caesarian sections defined 30 minutes as a standard for decision to delivery interval in a case of foetal distress, for an emergency delivery.[20] The need for general anaesthesia for foetal distress is controversial, with evidence supporting the use of regional anaesthesia in these cases.[21] If a functioning labour epidural is in situ, it can be used in all, but the most extreme cases may require administration of a rapidly acting local anaesthetic such as 3% 2-chloroprocain. The risk and benefit of general anaesthesia to neonates have been well-documented in several studies. Ong and colleagues[22] found that general anaesthesia was associated with higher incidents of lower Apgar scores as compared to regional anaesthesia. Gale and colleagues[23] reported that the number of neonates needing respiratory assistance at birth was twice as high in the group whose mothers receive general anaesthesia as compared to epidural anaesthesia. In a meta-analysis of anaesthesia for caesarean section, it has been concluded that spinal anaesthesia is not safer than epidural or general anaesthesia.[24] Bonnet *et al*. 25concluded that all Obstetric anaesthesia and analgesia techniques are associated with a theoretical risk of foetal distress, but given the fact that regional anaesthesia techniques are associated with a well-demonstrated benefit for the mother and new born, the latter remains the preferred choice in obstetric anaesthesia. The American College of Obstetricians and Gynaecologists has supported the use of regional anaesthesia in these cases.[26]

## CONCLUSION

It is concluded that a single maneuver of IUFR may be less effective than a combination, and there might even be potentiating between maneuvers and there is a problem of defining the outcomes, because of lack of out come studies. However, it helps to ‘buy time’ to optimize the foetal condition, while preparing for an impending delivery, or an emergency cesarean section, or as an interim strategy, while waiting for the caesarean section. The nature of an abnormal foetal heart rate dictates how long one can delay delivery or intervention before restoring to an emergency delivery. To assess the effectiveness of IUFR, rigorous randomized out come studies are required.
